# Sleep-related and non-sleep-related migraine: interictal sleep quality, arousals and pain thresholds

**DOI:** 10.1186/1129-2377-14-68

**Published:** 2013-08-06

**Authors:** Morten Engstrøm, Knut Hagen, Marte Bjørk, Gøril Bruvik Gravdahl, Trond Sand

**Affiliations:** 1 Department of Clinical Neurosciences, PB 8905, MTFS, Norwegian University of Science and Technology, Trondheim N-7489, Norway; 2 Department of Neurology and Clinical Neurophysiology, St. Olavs Hospital, Trondheim N-7006, Norway; 3 Norwegian National Headache Centre, St. Olavs Hospital, Trondheim N-7006, Norway; 4 Department of Neurology, Haukeland University Hospital, Bergen, Norway; 5 Department of Clinical Medicine, University of Bergen, Bergen N-5021, Norway

**Keywords:** Sleep, Arousal, Migraine, Sleep-related migraine, Non-sleep related migraine, Subjective sleep quality, Polysomnography, Pain thresholds

## Abstract

**Background:**

The mechanisms associating sleep and migraine are unknown. No previous polysomnographic (PSG) or pain-threshold (PT) study has compared patients with sleep-related migraine attacks (SM), non-sleep related migraine attacks (NSM) and healthy controls.

**Methods:**

We have performed a blinded, prospective exploratory study with case–control design. Thirty-four healthy controls, 15 patients with SM and 18 patients with NSM had interictal PSG heat-, cold- and pressure PT (HPT, CPT, PPT) recordings and completed diary- and questionnaire on sleep and headache related aspects.

**Results:**

NSM patients had more slow-wave sleep (SWS) and more K-bursts than SM patients (K-bursts: p = 0.023 and SWS: p = 0.030) and controls (K-bursts: p = 0.009 and SWS: 0.041). NSM patients also had lower HPT and CPT than controls (p = 0.026 and p = 0.021). In addition, SM patients had more awakenings and less D-bursts than controls (p = 0.025 and p = 0.041).

**Conclusion:**

SM- and NSM patients differed in objective-, but not subjective sleep quality. NSM patients had PSG findings indicating foregoing sleep deprivation. As foregoing sleep times were normal, a *relative* sleep deficit might explain reduced PT among NSM patients. The SM patients had signs of slightly disturbed sleep.

## Background

Approximately on third (24-42%) of migraine patients have attacks almost exclusively related to sleep or awakening (sleep migraine = SM) [1,2]. According to ICHD-II SM is not a separate migraine subtype [3], but sleep-related symptoms are among the most frequently cited trigger factors [4,5]. Sleep-related *headache* is defined by American Academy of Sleep Medicine (AASM) as complaints of headache (either migraine or other types) during sleep or upon awakening [6]. Sleep-related headache may also suggest the presence of an underlying sleep disorder [7,8].

Sleep disturbances are commonly reported by migraineurs [2,9,10]. However, it is not really known whether migraine attacks are the major cause of the reported sleep disturbances, whether coexistent sleep disturbances trigger migraine attacks during the night or if there are parallel (but non-causal) intrinsic pathophysiological mechanisms linking migraine and sleep problems [11,12]. One critical review article suggested that the significance of sleep as a migraine trigger still has not been conclusively determined [13]. Polysomnographic studies have been performed to detect sleep disorders and sleep characteristics in relation to migraine [14-16]; but the results are ambiguous. However, no previous study has compared sleep quality in patients with SM to patients with mainly non-sleep-related migraine attacks (non-sleep-related migraine = NSM). This comparison might clarify if disturbed sleep is a factor related specifically to nightly migraine attacks.

Furthermore, the relationship between pain mechanisms and sleep might contribute to explain why some people wake up with migraine. A relationship between sleep and pain has previously been found as sleep loss decrease pain thresholds (PT) in healthy people [17-20]. Disturbed sleep also seems to reduce the descending inhibitory pain control system (DNIC) activity and thereby increasing somatic spontaneous symptoms [21]. Decreased thermal PT (TPT) have also been found 24 hours before migraine attacks [22]. Hence we hypothesized that polysomnographic (PSG)-measures of sleep quality, nightly arousals and PT would be changed among SM patients compared to NSM patients and healthy controls.

Our primary aim of this exploratory, prospective case–control study was to compare subjective and objective sleep quality, arousal indices and PT between SM- and NSM patients. The second aim was to compare sleep and pain variables between healthy controls (C) and SM- and NSM patients respectively. Thirdly, we wanted to explore the correlation between subjective and objective sleep, PT, and headache severity variables.

## Methods

### Participants

Sixty-one migraine patients (M) with and without aura were recruited by advertising in local newspapers. Volunteers called a nurse trained in headache research for a screening interview followed by a consultation with a headache specialist who verified the diagnosis according to ICDH-II [3]. Subjects with two to six migraine attacks per month were selected for the present study. Subjects with coexisting frequent migraine and tension-type headache (TTH), other major health problems (including regular use of neuroleptic-, antiepileptic- or antidepressant drugs, analgesics, or drugs for migraine prophylaxis the last four weeks), or subjects who were pregnant, were not included. Painkillers or triptans for acute migraine were allowed. Based on the headache diary interictal (>two days from attack), patients were selected for the present study and the methodological details have been published previously [23].

The migraineurs were divided in two subgroups according to when the headache *usually started.* Migraineurs answering either “upon awakening” or “during the night (waking me up)” were defined as having SM, whereas those who answered “during daytime before noon”, “during daytime after noon” or “no regular onset time” were defined as having NSM. The pre- and postictal group were too small to qualify for a subgroup analysis. Thirty-three interictal migraineurs SM (n = 15) and NSM (n = 18) and thirty-four comparable controls according to age and sex were available for the analysis (Table 1). The study was approved by the regional ethics committee and participants signed an informed consent before inclusion.

**Table 1:** Baseline chracteristics and headache-related data: counts or mean (SD)

	***C (n = 34)***	***SM***^**1 **^***(n = 15)***	***NSM***^**1 **^***(n = 18)***
Age (years)	39.6 (13.7)	39.4 (14.3)	33.9 (11.4)
Sex: F/M	20/14	10/5	15/3
BMI (kg/m2)	**25.3 (3.4)**	23.6 (2.8)	**23.4 (3.4) **^**3**^
Caffeine beverages (cups per day)	**4.0 (3.5)**	2.7 (2.2)	**1.9 (2.4) **^**4**^
Alcohol: 0 (never) to 5 (4 or more per week)	**2.9 (1.0)**	2.3 (1.4)	**2.1 (1.1) **^**4**^
Nicotine: yes/no	6/28	2/13	3/15
Physical activity^2^	1.8 (1.2)	1.6 (0.9)	2.1 (1.4)
Days since last menstruation	18.9 (8.6)	16.0 (7.4)	13.3 (7.9)
Oral contraceptives: yes/no	7/13	5/5	5/10
Married or common-law partner/single	25/9	7/8	12/6
MA/MwoA		10/5	14/4
Headache time in diary (h/day)		1.7 (1.5)	2.0 (2.3)
Migraine time in diary (h/day)		0.8 (1.0)	1.8 (2.3)
Triptan days (0–14)		1.9 (2.3)	1.5 (2.4)
Analgesic days (0–14)		1.1 (1.6)	1.9 (2.3)
Headache days last 3 months		7.6 (7.6)	6.3 (3.3)
Headache intensity (1–4)		2.7 (0.5)	2.6 (0.5)
Migraine duration (years)		24.3 (17.7)	16.5 (12.0)
Photophobia (0–2)		1.1 (0.4)	1.1 (0.3)
Phonophobia (0–2)		0.9 (0.6)	1.1 (0.4)

Controls (C), SM: Sleep-related migraine attack group, and NSM: none-sleep-related migraine attack group.

^1^SM and NSM are the corresponding interictal subgroups (background data for interictal PSG and pain threshold variables reported in Table 3).

^2^(0: seldom), 1:1-2x/week, 2:3x/week; sum of exercise and walking to workplace scores.

^3^ NSM or SM ≠ C (p < 0.05). ^4^NSM or SM ≠ C (p <0.01).

Pearson chi-square test, Fisher exact test or Mann–Whitney U-test.

Significant differences are emphasized with bold types.

### Questionnaires and diaries

Headache hours per day, average sleep time, sleep latency, long (≥30 min) and short (<30 min) awakenings per night were calculated and analyzed from sleep diaries for the 14 days preceding the PSG.

Epworth sleepiness scale (ESS) [24,25], questions adapted from Karolinska sleep questionnaire (KSQ) [26], Pittsburgh sleep quality index (PSQI) [27] and Hospital anxiety and depression subscales (HADS) [28] were administered. The seven PSQI questions (indicating the frequency of common sleep problems; 0–3) were summed into a combined global score variable (PSQIgs, possible range 0–21) [27]. Bothersome tiredness was categorized (0–4) as “none”, “less than 7 days per month”, “7-14 days”, “> 14 days per month” and “daily”. We also had four questions concerning restless legs (“Urge to move the legs”, “Rest worsens the urge”, “Symptoms improve with movement”, “Symptoms worsen in the evening or night” [29]). Usual headache intensity, attack length, photophobia, phonophobia and migraine history duration were recorded from semi-structured nurse interviews (Table 1).

### PSG

Patients and controls underwent a full night sleep registration with ambulatory equipment. They slept unattended in our patient-hotel in the neighbor building. PSG was recorded by a Notta recorder (EEG Technology Int.bv, 6092 NM Leveroy, The Netherlands) and analyzed with Stellate Harmonie software (Stellate, Montreal, Quebec, Canada). Eight EEG electrodes were placed according to the International (10–20) system [30] (F3, F4, C3, C4, P3, P4, O1, O2 plus Pz reference and Cz ground); two electrooculographic electrodes (EOG) applied two cm lateral and respectively two cm up and two cm down from the right and left lateral eye cantus. EOG-reference electrodes were applied to the left (A1) and the right (A2) mastoids. Surface electromyography was registered from submentalis muscles, the left anterior tibialis muscle and trapezius muscle bilaterally.

The following sensors from Breabon Medical Corporation, Ontario, Canada were applied for respiration and circulation measurements: a three-point oronasal airflow thermistor (Airflow temperature sensor R-510), bands around thorax and abdomen to measure respiratory movements (Ultima Respiratory Effort Sensor™, piezo-electric crystals) and a body position sensor (Ultima Body Position Sensor™). An infrared index finger oxymeter (model 8000 J3, Nonin Medical Inc, Plymouth, USA) and 10 mm silver chloride cup ECG electrodes (Natus Medical Inc, San Carlos, USA) were also used. The participants were instructed to go to bed as usual, and write down light-off and light-on times using a synchronized wrist watch.

### PSG data analysis

Analyses were done from noted time for “lights off” in the evening to “lights on” in the morning. Respiratory events were scored automatically and edited visually later. The AASM manual suggest 2 hypopnea definitions based on nasal pressure signals but we had a thermistor. Therefore we chose to analyze hypopneas according to a modified “Chicago criteria” [31] (≥50% amplitude reduction or lower amplitude reduction in thermistor signals associated with 4% desaturation). Automatic periodic leg movement (PLM) analysis was implemented according to the AASM criteria [32]. Manual sleep scoring, arousal scoring and event editing were performed by the first author (specialist in clinical neurophysiology assisted by a sleep expert (last author)). Sleep staging was performed according to “The AASM Manual for the scoring of sleep and associated events” from 2007 [32] with a few exceptions, as described below.

In the present study we wanted to explore fast arousals besides slow arousals as separate events without considering the time interval between the different episodes. Since low frequency episodes have been defined and scored separately as arousals before [33], these D- and K-burst events were used as the “slow counterpart” to the fast AASM-arousals.

First, fast arousals were defined according to the AASM-manual [32] as an abrupt shift of EEG frequency (alpha, theta and/or faster than 16 Hz activity) lasting 3–30 seconds, separated with at least 10 seconds of sleep. Arousals were scored in NREM and in REM sleep if associated with increased EMG for more than one second. Although the upper limit for arousal definition is not defined by AASM, we chose to use 30 s in the present study to avoid ambiguous counts induced by random timing of sleep staging epochs. In this way, an e.g. 25-second EEG-frequency increase will always be counted as one arousal event regardless of its relationship to the epoch boundaries. Therefore, only changes in EEG-activity containing dominating frequencies of 8 Hz or more lasting more than 30 seconds were classified as an awakening. If a sleep stage N2 K-complex was followed by a high frequency arousal, we scored the arousal without changing the sleep stage to N1.

Secondly, we also scored two additional PSG measures of slow-wave arousal: Delta-bursts (D-bursts), defined as a sequence of delta waves lasting 2 s or more and exceeding the background amplitude with at least one third [33], and K-bursts, defined as at least two consecutive K-complexes [33]. A K-complex is a negative deflection followed by a positive component with a minimum duration of 0.5 seconds and minimum peak to peak amplitude of 75 μV. Both fast and slow arousals were scored when observable in at least three out of eight EEG channels. Awakening-, arousal- K- and D-burst- indexes were calculated as event number per sleep hour. Since K- and D-bursts probably reflect similar physiological processes [34,35] they were combined into a KD-index (resembling Cyclic Alternating Pattern (CAP) A1 phase) for correlation analyses in the present paper. The combined KD-index was also chosen rather than the individual K- and D-burst indexes to reduce the amount of analyses.

### Pain thresholds (PT)

Thermal PT (TPT) and pressure PT (PPT) (algometry) were recorded one hour before the participants had their PSG equipment mounted. Heat and cold PT (HPT and CPT) were measured separately in a fixed order on thenar and the medial forehead on both sides with methods of limits (Senselab – thermotest, thermode area 25× 50 mm^2^, Somedic Sales AB, Sweden). Temperature was increased by 1°C/s from a 32°C baseline to a 50°C maximum for three HPTs followed by three decreasing temperature stimuli to 5°C minimum for CPT. PPT were measured at four sites on both sides in a fixed order: m. temporalis (10 mm lateral to the external angle of the orbit), m. splenius (C2 level just at the edge of the trapezius muscle about 35–40 mm lateral to the midline), m. trapezius (10 mm lateral to the midpoint of a line connecting the acromion and the spinous process of C7) and over distal phalanx middle finger (Algometer type II, probe area 1 cm^2^, Somedic Sales AB, Sweden). Pressure was increased with 30 kPa/s. Thresholds were repeated three times, left before right, and the average was calculated. All thresholds were measured by one out of two technicians. In subjects who did not feel cold pain at 5°C, we used the substitution value 4°C. TPT were expressed as differences from baseline: HPTd (HPT-32) and CPTd (32-CPT) and averaged (right and left sides from all recorded sites) for the present analysis.

### Blinding

For the PSG and PT measurements the technicians were blinded for diagnoses. Scoring of the PSG data was also performed blinded for diagnoses. Two nurses administered the participant appointments and questionnaires. They also accompanied the participants to the technicians after having instructed the participant not to tell anything that could reveal their headache trait or state.

### Statistics

Statistical analyses were performed with PASW statistics v.18 and SYSTAT version 11. Since we found no significant differences in sleep-variables between migraine with and without aura, migraine patients were analyzed as one combined group in the present study.

Two-group comparisons were made by nonparametric Mann–Whitney tests. Categorical data were analyzed with Pearson chi-square test or Fisher’s exact test if any cross tabular cells had expected count less than five.

Two-sided p-values less than 0.05 were regarded as significant. No adjustments for multiple comparisons were done because the study was exploratory and we wanted to avoid excessive type II errors and to avoid inappropriately testing of a less relevant universal null-hypothesis [36].

Partial correlation coefficients (adjusted for age) were calculated to explore the association between migraine severity, PT, sleep quality, arousal and sleep symptoms in SM- and NSM patients. Variables were square-root transformed before this calculation.

The power (based on Student’s t-tests) to detect large effect sizes equal to 0.8 (and 0.9) SD in two-group comparisons were 78% (87%) for C - NSM, 73% (83%) for C – SM and 63% (73%) for SM – NSM.

## Results

### Baseline characteristics and headache-related data

There were no differences between SM- and NSM patients in baseline characteristics or headache data (Table 1). NSM patients had lower BMI and consumed less caffeine and alcohol than controls, and both NSM- and SM patients had more anxiety symptoms than controls. Triptans were taken within 48 hours before the interictal PSG by two SM- and three NSM patients.

### Sleep related symptoms

Self-reported sleep related symptoms were not significantly different between SM- and NSM patients (Table 2). Both migraine groups reported more subjective sleep problems regarding insomnia, global sleep problems (PSQI) and pain-related sleep difficulties compared to controls. NSM patients were also more often subjectively tired than controls. However, there were no significant differences in hypersomnia as measured by ESS.

**Table 2:** Sleep diary, sleep disorder symptoms, autonomic instability symptoms and emotional state

	**C (n = 34)**	**SM (n = 15)**	**NSM (n = 18/17**^**10**^**)**
Average diary sleep time (hour)	7.3 (0.8)	7.2 (1.0)	7.3 (0.9)
Long awakenings in diary^1^ (no)	0.1 (0.2)	0.2 (0.2)	0.3 (0.5)
Short awakenings in diary^2^ (no)	0.2 (0.2)	0.2 (0.5)	0.3 (0.5)
Sleep latency in diary^3^	0.4 (0.4)	0.7 (0.8)	0.5 (0.5)
Epworth sleepiness scale (0–24)	5.6 (3.1)	5.5 (3.3)	7.2 (4.7)
Snoring/apnea KSQ score (0–8)	1.7 (1.6)	1.5 (1.3)	1.6 (1.8)
Daytime tiredness frequency (0–4)	**0.7 (0.8)**	1.1 (1.0)	**1.3 (1.0) **^**12**^
Insomnia KSQ score (0–16)^4^	**3.4 (2.3)**	**6.3 (3.7) **^**12**^	**5.8 (2.8) **^**12**^
PSQIgs (0–21)^5^	**3.8 (2.6)**	**6.5 (3.1) **^**12**^	**5.9 (3.5) **^**11**^
Pain-related sleep trouble (1–4)^6^	**1.3 (0.7)**	**1.8 (1.0) **^**11**^	**2.0 (1.1) **^**12**^
Restless legs (0–1)^7^	0.1 (0.4)	0.3 (0.5)	0.3 (0.5)
Depression score (0–21)^8^	1.6 (2.1)	2.1 (2.1)	2.9 (2.8)
Anxiety score (0–21)^8^	**2.9 (2.6)**	**5.3 (2.6) **^**12**^	**6.0 (3.3) **^**12**^
Autonomic index (0–30)^9^	**1.5 (1.4)**	**5.7 (5.0) **^**13**^	**6.3 (3.3) **^**13**^

Controls (C), SM: Sleep-related migraine attack subgroup. NSM: non-sleep-related migraine.

^1^Awakenings lasting more than 30 minutes.

^2^Awakenings lasting less than 30 minutes.

^3^Categorized as 0: <15 min, 1: 15–30 min, 2: 30–90 min, 3: > 90 min.

^4^Sum of four insomnia-questions in KSQ (Karolinska sleep questionnaire).

^5^Pittsburgh sleep quality index.

^6^frequency of pain-related sleep problems during the last month from the PSQI questionnaire. ^7^Restless legs (1 = answered yes to all four obligatory criteria).

^8^Sum of seven questions about depression and anxiety symptoms respectively during the last week from the Hospital Anxiety and Depression Scale (HADS) questionnaire.

^9^Sum of 10 questions about palpitations, nausea, unsteadiness, muscle weakness, tremulousness, dizziness, blurred vision, shivering, and paleness.

^10^Diary data lacking from one NSM subject.

^11^NSM or SM ≠ C (p < 0.05). ^12^ NSM or SM ≠ C (p <0.01). ^13^ NSM or SM ≠ C (p <0.001). (Mann–Whitney U-test). Significant differences are emphasized with bold types.

### Polysomnographic sleep quality, arousals and PT

NSM patients had more slow wave sleep (SWS, stage N3, p = 0.023), more K-bursts (p = 0.030) and slightly higher nightly mean SaO2 than SM patients (Table 3). A slightly higher awakening index (p = 0.025), lower D-index (p = 0.04) and a tendency to more superficial stage N1 sleep were found among the SM patients compared to controls (p = 0.05). Fast arousal index among the SM patients did not differ from controls either judged by the whole night or separated into specific NREM or REM indexes (not tabulated). There were no significant differences in sleep efficiency, sleep-onset latency, or REM latency.

**Table 3:** PSG sleep quality, and pain threshold mean values (SD) for controls, and interictal migraine subgroups

	**C (n = 34)**	**SM (n = 15)**	**NSM (n = 18)**
Time in bed	**453 (58)**	465 (57)	**488 (46)**^**4**^
Total sleep time (min)	409 (68)	417 (67)	451 (52)
Sleep efficiency (%)	90.0 (8.1)	89.4 (7.6)	92.4 (4.2)
Latency to sleep onset (min)	12.8 (14.6)	13.8 (25.1)	7.4 (7.7)
Awakening index (no/h)	**0.99 (0.59)**	**1.45 (0.84)**^**4**^	1.12 (0.63)
Wake after sleep onset (min)	30.9 (26.7)	34.5 (19.0)	29.5 (16.4)
Stage N1 (min)	**27 (19)**	**35 (17)**^**6**^	29 (13)
Stage N2 (min)	197 (47)	194 (44)	206 (45)
Stage N3 (min)	**86 (31)**	**88 (25)**^**3**^	**104 (28)**^**3,5**^
REM (min)	99 (26)	99 (38)	112 (32)
SaO2 mean (%)	**95.2 (1.4)**	**95.2 (1.4)**^**3**^	**96.1 (1.0)**^**3,4**^
Apnea-hypopnea index (per hour)	2.7 (3.3)	2.6 (2.6)	2.2 (3.3)
Periodic limb movement index (per hour)	6.7 (10.3)	4.0 (6.3)	8.4 (11.4)
Fast arousal index (per sleep hour)	**18.3 (5.7)**	17.4 (8.6)	**15.5 (9.7)**^**4**^
Fast arousal index (per hour REM sleep)	21.8 (8.7)	21.3 (11.3)	18.2 (14.6)
KD-burst index (per sleep hour)	14.8 (10.9)	9.6 (7.3)	15.4 (9.8)
D-burst index (per sleep hour)	**11.8 (8.0)**	**7.3 (5.7)**^**4**^	11.3 (8.3)
K-burst index (per sleep hour)	**3.0 (3.8)**	**2.4 (2.2)**^**3**^	**4.0 (2.5)**^**3,4**^
PPTavg^1^ (kPa)	**661 (249)**	586 (141)	**519 (125)**^**6**^
HPTavg^2^ (°C)	**13.6 (3.1)**	12.6 (3.3)	**11.2 (3.7)**^**4**^
CPTavg^2^ (°C)	**20.7 (6.3)**	18.3 (6.3)	**16.1 (7.4)**^**4**^

C: Controls, SM: Sleep-related migraine attack subgroup and NSM: non-sleep-related migraine recorded in an interictal phase. PPT: pressure pain threshold, HPT: Heat pain threshold, CPT: cold pain threshold. avg: Regional averages from either ^1^splenius, trapezius, temporalis, and index finger or ^2^forehead and palm. HPT and CPT are expressed as differences from the 32°C baseline.

Mann–Whitney U-test: ^3^NSM ≠ SM (p < 0.05). ^4^NSM or SM ≠ C (p < 0.05). ^5^NSM ≠ C (p <0.01), ^6^NSM or SM ≠ C (p = 0.05). One control and two NSM subjects were excluded from the PLM analysis and one SM from arousal analysis because of loosened electrodes. Significant differences are emphasized with bold types.

NSM patients spent more time in bed (p = 0.041), had more stage N3 slow-wave sleep (p = 0.009) and a lower index of fast arousals (p = 0.041) than controls. TPT were lower among the NSM patients than controls (p = 0.026 and p = 0.041). PPT also tended to be lower among the NSM patients compared to the SM patients (p = 0.08).

### Partial correlations controlled for age in NSM and SM and controls

**Figure 1:**
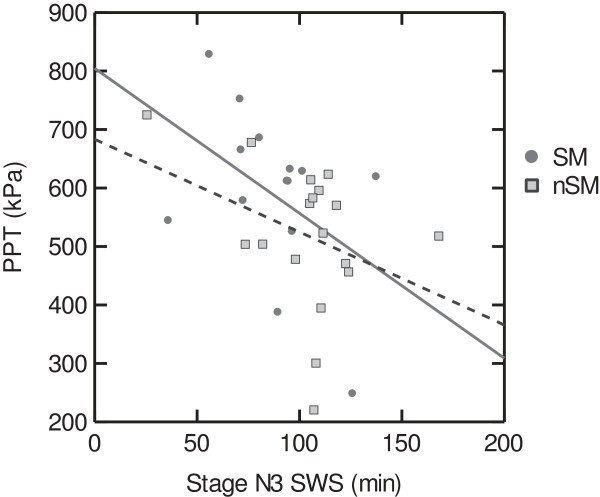
**Pressure pain thresholds and amount of slow wave sleep (SWS) in sleep related migraine (SM, solid line) and non-sleep related migraine (NSM, dotted line).** Less N3 sleep was significantly associated with high pressure pain thresholds in the NSM group (age adjusted r = -0.50. p < 0.05).

**Figure 2:**
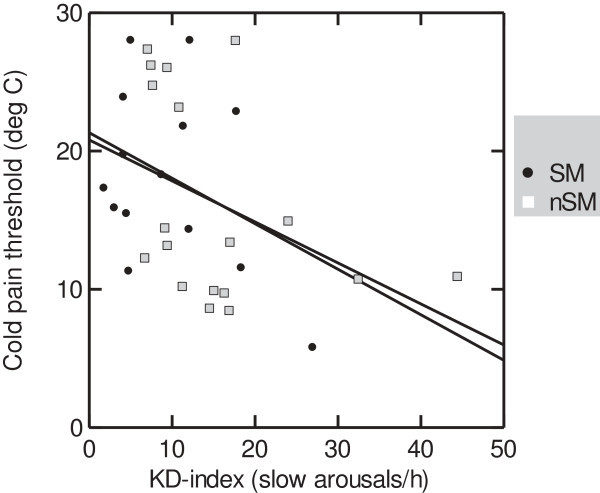
**Cold pain thresholds and D-burst index in sleep related migraine (SM, solid line) and non-sleep related migraine (NSM, dotted line).** Cold pain threshold is expressed as the difference from baseline. KD-bursts are significantly associated with cold hyperalgesia (age-adjusted r = -0.59, p < 0.01).

Among NSM patients the amount of sleep stage N1 correlated positively with headache frequency (Table 4). The amount of N3 sleep correlated negatively with PPT (Table 4, Figure 1). The amount of KD-bursts correlated negatively with TPT (Figure 2) and headache frequency (Table 4). Among SM patients, the KD-index tended to correlate positively with PPT. The amount of N2 sleep correlated negatively with insomnia and positively with PPT. The amount of sleep stage N3 correlated negatively with headache history duration. Among controls we found no significant correlations in objective versus subjective quality scores or versus PT (not tabulated).

**Table 4:** **Partial age**-**adjusted correlations**^**1 **^**between sleep**, **symptoms**, **pain thresholds**, **among interictal migraineurs subgroups**

	**Sleep quality**		**Pain thresholds**			**Headache**		
**NSM (n = 18)**	**Insomnia**^**2**^	**PSQI**^**3**^	**PPT**	**HPT**	**CPT**	**Headache days**^**4**^	**Headache intensity (1–4)**	**Headache duration (years) **^**1**^
Sleep N1	0.12	0.41	0.16	0.02	0.16	0.60*	0.21	0.35
Sleep N2	0.18	0.26	0.29	-0.05	-0.04	0.18	0.08	-0.11
Sleep N3	-0.40	-0.47(*)	-0.50*	-0.18	-0.21	-0.24	0.03	-0.23
REM sleep	-0.15	-0.25	-0.04	0.08	-0.27	0.21	0.19	0.03
Fast arousal index	0.46(*)	0.45(*)	0.30	-0.19	-0.23	-0.34	-0.10	0.09
Slow arousal (KD-) index	0.13	-0.33	0.05	-0.57*	-0.57*	-0.64**	-0.01	-0.46(*)
**SM (n = 15**^**5**^**)**	**Insomnia**^**2**^	**PSQI**^**3**^	**PPT**	**HPT**	**CPT**	**Headache days**^**4**^	**Headache intensity (1–4)**	**duration (years)**
Sleep N1	0.32	0.28	0.07	-0.12	0.08	0.11	0.19	-0.13
Sleep N2	-0.69**	-0.46(*)	0.54*	-0.20	0.08	-0.50(*)	0.04	0.28
Sleep N3	0.41	0.33	-0.36	-0.11	-0.44	0.39	-0.22	-0.63*
REM sleep	-0.13	-0.11	0.15	0.10	0.34	-0.40	-0.01	-0.06
Fast arousal index	0.30	0.26	0.15	-0.21	-0.08	-0.02	0.18	-0.53(*)
Slow arousal (KD-) index	-0.36	-0.01	0.53(*)	-0.19	-0.31	0.05	0.40	-0.12

NSM: Non-sleep related migraine. SM: sleep related migraine.

^1^All variables were square root transformed before correlation was computed. SM: Sleep-related migraine attack subgroup and NSM: non-sleep-related migraine recorded in an interictal phase. PPT: pressure pain threshold, HPT: Heat pain threshold, CPT: cold pain threshold.

^2^Sum of four insomnia-questions in KSQ (Karolinska sleep questionnaire).

^3^Pittsburgh sleep quality index frequency of pain-related sleep problems during the last month from the PSQI questionnaire.

^4^Headache days last three months.

^5^One SM excluded from fast and slow arousal scoring because of loosened electrode.

p < 0.10(*), p < 0.05*, p < 0.01** Significant differences are emphasized with bold types.

## Discussion

In this first prospective exploratory case–control study evaluating NSM- and SM patients and controls, we found that NSM had increased objective sleep quality, increased subjective daytime tiredness and reduced PT. The SM patients on the other side had signs of reduced objective sleep quality, but had normal PT and did not report increased subjective daytime tiredness.

### NSM

NSM patients had increased daytime tiredness, more SWS (N3) sleep, less fast arousals and a higher K-burst index than controls. After sleep deprivation in healthy controls increased amount of K-complexes [37] and SWS [37,38] and reduced amount of fast arousals [38] are found. However, based on the sleep diaries the NSM patients had the same sleep times as the SM patients and controls. A higher need of sleep and a relative sleep deprivation among the NSM patients could be one explanation. Another explanation could be hypoarousability, i.e. hypofunction of the CNS arousal system [14].

The lower caffeine intake among NSM patients can possibly be related to the daytime tiredness. However, reduced caffeine intake is unlikely to explain our PSG findings among NSM patients because a low-to-moderate amount of caffeine has been found to have little effect on subjective and objective sleep apart from more stage I sleep (Rechtschaffen and Kales) in insomniacs [39].

We also found reduced TPT among the NSM patients. Reduced PT has previously been found among sleep-deprived healthy persons [17-20]. In addition, EEG has in several old studies been found to resemble drowsiness in several migraineurs [40]. These observations do also fit with our interpretation of the present data, i.e. that the NSM patients may be hypoaroused and suffer from (relative) sleep deprivation.

Moreover, there were negative correlations between N3 sleep and PPT and between KD-bursts and TPT among the NSM patients. Slow bursts are said to reflect the slow wave sleep propensity, occurring with highest frequency before SWS in the first sleep cycles [41]. Hence, increased SWS and slow bursts could indicate a higher need for sleep in NSM patients compared to SM patients. Our findings do also suggest a possible dose–response relationship between the proposed sleep deprivation and the reduced PT.

### SM

The SM patients had no signs consistent with sleep deprivation (e.g. increased daytime tiredness and reduced PT) even though we found signs of disturbed sleep compared to controls. Age-adjusted headache duration in years was related to a distinct reduction of SWS among SM patients. Also, increasing insomnia symptoms were mainly related to reduced N2 sleep and there was an association between reduced N2 sleep and reduced PT. Furthermore SM patients also had fewer K-bursts than NSM patients. In coherence with findings of Della Marca [14] SM patients also had fewer D-bursts than controls. Slow bursts are frequent before and during SWS [41]. If slow bursts can be interpreted as a measure of the ability to get enough SWS, it also is consistent with a reduced ability to achieve sufficient SWS among SM patients. N2 sleep then might partially compensate for a possible lack of SWS in SM patients.

Nightly hypoxia is related to headache and tiredness [11] and SM patients had a slightly lower mean SaO2 during the whole night than NSM patients. However, mean SaO2 did not differ in SM patients compared to controls, and we could not detect any significant difference in the apnea- hypopnea index between these groups.

### What is the importance of headache onset time?

In our first paper from this study [23], interictal migraineurs had increased amount of awakenings during sleep, but paradoxically tended to have less fast arousals and more slow wave sleep. From the present results we can see that the explanation is subgroup differences. The SM patients had increased awakenings and the NSM patients had increased slow wave sleep and less fast arousals.

A migraine attack may be interpreted as an example of genetically determined adaptive behavioral response to internal or external stressors that it is orchestrated by a threatened brain [42]. Also in the present study both SM and NSM had increased “load” with more symptoms of sleep disturbances and anxiety compared to controls [43,44]. Increased neural activity can increase sleep need [45], but we could not detect differences in work frequency, physical activity, affective or sleep symptoms between the NSM and SM groups.

Why were the SM patients not equally or more tired than the NSM patients? In line with Cortelli [42] we found signs of slightly disturbed sleep among SM patients and signs indicating that daytime is more tiring for NSM patients than controls. It can be hypothesized that the lack of response decrement to repeating stimuli (habituation) described among migraineurs [46] might chiefly be a characteristic sign of NSM patients.

The apparently preserved arousability among SM patients might in fact be sign of high resistance towards daytime load consistent with the idea that SM patients do not reach their overload limit before night-time. The negative effect of high arousability might be an increased vulnerability to reduced sleep quality in SM patients. It is possible that small disturbances in sleep quality (that normally are not perceived as sleep disruptive) are the drops that makes the flood in these patients; i.e. triggering a migraine attack during night. This notion fits with the parallel increase in sleep disturbances and sleep related migraine with age [47] and with the proposal that apnea disrupted sleep also can trigger a migraine attack [48].

The “hypoarousability” of the NSM and robust arousability among SM could in principle be related to the periaqueductal gray matter, a structure which is assumed important in the migraine pathogenesis [49], since “hypoarousal” and “hyperarousal” has been linked to ventrolateral and lateral regions respectively [50].

### Strengths and limitations

The strength of this study is the blinded, controlled, prospective, and population based design, thereby also avoiding hospital-based severe and longstanding migraine cases.

There are different arousal definitions: AASM accepts only fast arousals [32]. Phase of transient activation (PAT) [33] is another definition of fast arousals. Low frequency episodes as D- and K-bursts are found related to temporarily increased heart rate and scored separately as arousals before [33]. The CAP system includes beneficially both fast and slow arousals in one scoring system [51] and is also previously used in examination of sleep among sleep-migraineurs [14]. However, CAP scoring covers only NREM sleep, is quite complicated to score [52] and sleep- migraineurs’ sleep is previously also investigated without CAP scoring [15]. In the present exploratory study we included both fast and slow arousals as in the CAP system, but we intended to score both fast and slow arousals separately, without considering sleep phase and the time interval between the different arousal episodes.

Only one PSG recording from each patient in the present study excluded the possibility to evaluate intra individual changes in different migraine phases. A possible first night effect on slow D- and K-arousal bursts and fast microarousal bursts was non-significant and very small in one study [53]. Besides, any other systematic first night-effects would probably affect groups in a similar way and accordingly be cancelled out in a statistical comparison. Our method for separating migraineurs into SM- and NSM patients by one question on the most typical headache onset time is a weakness. A more objective way would be to evaluate headache onset in diaries for several months. However, as we only had diaries for four weeks, this alternative was not possible in the present study.

Furthermore no corrections for multiple comparisons were done because the study was exploratory, and we did not want to increase type II failures on the cost of reducing type I failures [36,54]. However, the chosen approach does increase the risk of false positives (type I errors) and our findings should accordingly be independently reproduced before firm conclusions can be drawn.

## Conclusion

In conclusion, in this first prospective exploratory case–control study evaluating sleep in interictal SM- and NSM patients and controls, we found small, but probably important differences. NSM patients showed a sleep pattern consistent with foregoing sleep deprivation even if sleep times in sleep diaries were normal. SM patients on the other hand, had signs of slightly disturbed sleep. As far as we know, no others have compared PSG-sleep between NSM and controls. However, our results need independent confirmation.

## Competing interest

The authors declare that they have no competing interest.

## Authors’ contribution

ME mounted some PSGs and performed some pain threshold measurements, analyzed all PSGs, performed the statistical analysis, prepared the initial draft and was the main author of the present manuscript. KH included patients in the study. GG was contact person for the participants, handled and typed all questionnaires. TS had the original idea of the study; he has made all the data files for statistics and been the main supervisor in all processes. All authors have contributed to the practical plans for the study, read, revised and approved the final manuscript.
